# Flexible, Heat-Durable, and Highly Sensitive Piezoelectrets from Cyclic Olefin Copolymer with Microhoneycomb Structure

**DOI:** 10.3390/mi14040829

**Published:** 2023-04-08

**Authors:** Hui Wang, Xiaolin Wang, Changchun Zeng

**Affiliations:** Department of Industrial and Manufacturing, FAMU-FSU College of Engineering, High-Performance Materials Institute, Florida State University, Tallahassee, FL 32310, USA

**Keywords:** piezoelectrets, cyclic olefin copolymer, electromechanical property, piezoelectric sensitivity, pressure sensing

## Abstract

This paper discusses the fabrication and characterization of cyclic olefin copolymer (COC)-based pseudo-piezoelectric materials (piezoelectrets) with exceptionally high piezoelectric activity, and their potential use in sensing applications. Piezoelectrets that utilize a novel microhoneycomb structure to achieve high piezoelectric sensitivity are carefully engineered and fabricated at a low temperature using a supercritical CO_2_-assisted assembly. The quasistatic piezoelectric coefficient *d*_33_ of the material can reach up to 12,900 pCN^−1^ when charged at 8000 V. The materials also exhibit excellent thermal stability. The charge build-up in the materials and the actuation behavior of the materials are also investigated. Finally, applications of these materials in pressure sensing and mapping and in wearable sensing are demonstrated.

## 1. Introduction

Nontoxic, flexible, highly sensitive, and low-cost pressure sensors are crucial in the development of wearable or implantable measuring devices. A large variety of such sensors have been designed and developed based on different sensing mechanisms, such as capacitive, piezoresistive, and piezoelectric properties [1]. Utilizing the piezoelectricity of the sensing materials, piezoelectric pressure sensors produce electrical signals when pressure is applied. Currently, the most widely used piezoelectric material is ceramic-based piezoelectric material, such as PZT ceramics. Polymer piezoelectric foams (piezoelectrets) are porous polymeric materials with internally charged polymer pores (artificial voids or cavities). The charged pores are macroscopic electrical dipoles, which give rise to the piezoelectric behavior. As the piezoelectricity is not of molecular origin, pseudo-piezoelectricity is commonly used to refer to this piezoelectric-like behavior. Since they were first investigated in Finland in 1989 [2], these materials have attracted considerable attention because of their advantages in several important areas, such as being flexible, nontoxic, lightweight, low-cost, and high in piezoelectric activity [3], as compared to traditional piezoelectric materials. Prepared from a wide variety of polymers [4,5,6,7,8,9,10,11], they have been demonstrated to have potential in pressure-sensing [12], actuating [13] and energy-harvesting applications [14].

The performance of the piezoelectrets depends on both the properties of the polymers and the porous structure of the foams. In particular, the thermal stability is dictated by the polymer used. For example, the most commercialized working piezoelectric foam, polypropylene piezoelectric (PP) foam, only has a working temperature of approximately 70–80 °C [15,16].

A variety of polymers with higher thermal stability have been explored for use in piezoelectret fabrication [5,7,8,11,17,18]; however, they either showed low piezoelectric activity because of their less-than-optimal porous structure, sacrificed structural stability, or both. For example, fluoropolymers, known for their outstanding thermal stability, suffer from severe creep behavior, which limits their piezoelectret applications. The piezoelectric activity, one of the most important properties, is largely determined by the porous structure. Although a variety of porous structures were produced using different fabrication methods [17,19,20] and high piezoelectric activity was realized in some materials [4], piezoelectrets with high piezoelectric activity and high thermal stability are lacking. This is largely due to the lack of suitable fabrication methods to realize the ideal ‘disk-like’ pores [4,21] from polymers with excellent thermal stability, charge storage capability, and mechanical properties. 

This work studied the design, fabrication, and characterization of cyclic olefin copolymer (COC) piezoelectrets of high piezoelectric activity. COC is a polymer with excellent thermal and environmental stability, outstanding mechanical and dielectric properties (low dielectric constants and dielectric losses) [22], and excellent charge storage capabilities [23]. Several studies have shown that piezoelectric foams of COC demonstrate considerable thermal stability [23,24,25,26]. The porous structure in the study was based on a honeycomb design mimicking the ideal cellular morphology for high piezoelectricity. We showed that the piezoelectric activity of the material can be as high as 12,900 pCN^−1^. To the best of our knowledge, this is among the highest piezoelectric sensitivity ever achieved in this type of pseudo-piezoelectric material from any polymer. The thermal stability of the charged structure was studied using thermally stimulated discharge, and the piezoelectrets remained piezoelectrically active at a temperature of 150 °C. The charge build-up process inside the artificial void was investigated using an electric hysteresis loop, and the actuation behavior was studied using a butterfly loop. Finally, sensor devices were fabricated using COC piezoelectret sensors and sensor arrays, and their capabilities in impact detection, pressure sensing, and pressure mapping were demonstrated.

## 2. Result and Discussion 

### 2.1. Design Rationale

In general, the piezoelectric coefficient is related to the surface charge density σ and the elastic modulus Y of the porous material by the following relationship [27,28]:

(1) $$d_{33}\propto \frac{\sigma }{Y}$$

The porous structure of the piezoelectrets has a profound influence on the elastic modulus of the porous materials, while the charging condition mainly affects the surface charge density inside the artificial void. A reduction in the modulus of the structure would lead to improvements in its piezoelectric activity.

The elastic modulus of the material is primarily affected by the porosity and aspect ratio of the pores (artificial voids) by the following relationship [29]:

(2) $$Y\propto \phi^{2}(\frac{d}{L})$$

where ϕ is the porosity of the material and *L*/*d* is the aspect ratio of the artificial void. It follows that a high porosity and high aspect ratio are desired for a low elastic modulus and high $d_{33}$ Unfortunately, for most thermally stable polymers, achieving high porosity is difficult with existing technology, and obtaining a high aspect ratio with good controllability is even more challenging. For example, direct foaming typically results in isotropic pores and can only achieve low-to-moderate porosity [30]. For instance, extrusion foaming was used for COC and the prepared piezoelectrets had a very low $d_{33}$ of ~50 pCN^−1^ [23]. The thermal fusion of preformed cavities improved overall porosity in Ref. [5], but accurate control of the high aspect ratio was still challenging. 

Previously, we utilized a design that consisted of offsetting ridges and cavities to increase the porosity and enable a certain degree of control over the aspect ratio of COC piezoelectric foams [4]. As a result, a good $d_{33}$ of ~1300 pCN^−1^ was achieved. However, such a design has limitations in the range of porosity and aspect ratio that can be achieved, as a too-high porosity and aspect ratio would lead to structure instability. 

The honeycomb design utilized in this study overcomes these major limitations and allows for significantly higher porosity Φ. It also provides better control of the aspect ratio and more error tolerance through controlling the structure parameters a, b, and t of the honeycomb structure. The width of the hexagon ‘a’ can be controlled by using a metal mold with patterns of high precision, while the hexagon height ‘b’ can be controlled through adjusting the total thickness of the assembled structure using a high-precision clamp with micrometer precision. The elastic modulus can be further tailored by using films of different thickness ‘t’. In short, such a design allows for a robust fabrication of piezoelectrets with significantly higher piezoelectric activity with greater consistency. To keep the number of fabricated structures within a reasonable range, a single hexagon width of a = 3000 µm was used for this study.

### 2.2. Morphology

**Figure 1:**
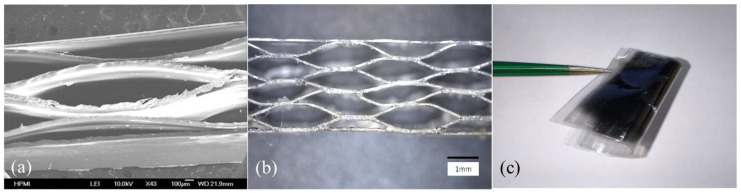
(**a**,**b**). SEM and optical microscope image of cross-section of the honeycomb structure; (**c**). Photograph of prepared flexible COC piezoelectric sensor (4 layers of COC film).

Figure 1a,b show the SEM and optical image of the cross-section of the fabricated COC piezoelectrets. The structure consisted of a highly ordered honeycomb microstructure with rather uniform pore structures. Despite the smooth edges between the sides of the hexagon unit structure, the honeycomb-like shapes were largely preserved. The layers were well bonded together with no apparent disjoining between the layers. Overall, the assembled structure conformed to the design with good structural fidelity. The piezoelectret was quite flexible and could withstand substantial bending (Figure 1c).

The fabrication of the honeycomb structure with good structural control was enabled by a novel supercritical CO_2_-assisted assembly. Being highly thermally stable with a glass transition temperature of 178 °C, COC 6017 typically requires high temperatures to process, leading to potential material oxidation. We utilized the unique characteristics of a COC–CO_2_ system to realize the fusion of the layer and the assembly of the structure at 120 °C, ~60 °C below the glass transition temperature of the material. This approach [4] leverages the substantial CO_2_ solubility in COC and the resultant reduction in the glass transition temperature of the polymer, as well as increased polymer chain mobility [31,32] to realize fusion between the layers at a significantly lower temperature. Moreover, guided by thermodynamic analysis [33], a temperature slightly lower than the COC–CO_2_ bulk glass transition temperature (120 °C) was chosen to bond the structure, utilizing the understanding of a more severe glass transition temperature depression and greater chain mobility at the polymer surface [34]. This enables the molecular-scale interfacial wetting, diffusion, and randomization to form bonding at the interface, while preserving the designed structure in the bulk. This is particularly important for the successful fabrication of a large-aspect-ratio pore structure and for preserving high porosity, as the thermal bonding used in other studies [5,35,36], that relied on the partial melting of the materials to form a bonded interface, would inevitably deform and collapse the pores, partially or completely destroying the designed structure. 

### 2.3. Effect of Design Parameters on Piezoelectric Coefficient

**Figure 2:**
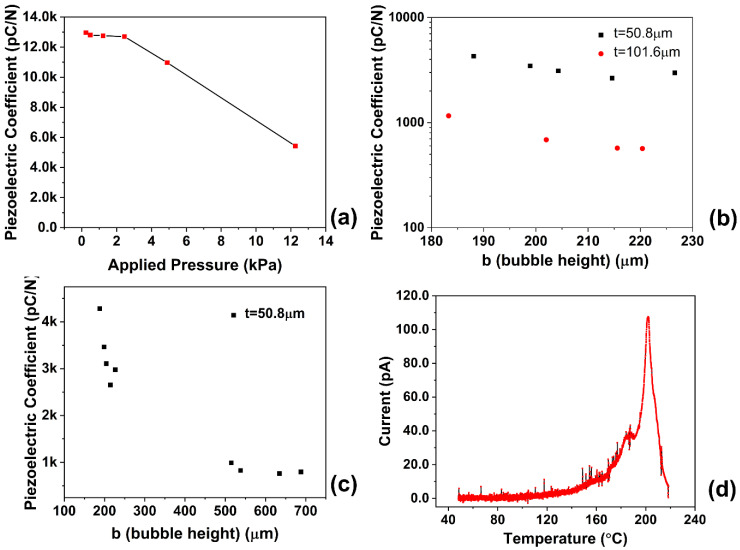
(**a**). Quasistatic piezoelectric coefficient of prepared piezoelectric foam (b = 240 μm, t = 50.8 μm); (**b**). Foam wall thickness’s effect on the sensitivity of the material; the d_33_ value was under a pressure of 4.9 kPa; (**c**). Artificial void height’s effect on the sensitivity of the material; (**d**). Thermally stimulated discharge of prepared piezoelectric foam (b = 240 μm, t = 50.8 μm).

Figure 2a shows the quasistatic piezoelectric coefficient of a piezoelectric foam with a 50.8 μm thick wall and a bubble height of 240 μm. The piezoelectret showed exceptionally high piezoelectric activity. A piezoelectric coefficient of 12,950 pCN^−1^ was obtained at low pressures and could remain at 6000 pCN^−1^ at higher pressures. This is an extremely high piezoelectric coefficient compared to other piezoelectric foams and previous COC-based piezoelectric materials, as in most of the recent work, $d_{33}$ was reported to be around 500–1500 pCN^−1^ at this pressure range. At a low pressure range, the piezoelectric coefficient was independent from the pressure. The material was in its linear elastic deformation region. The decreased $d_{33}$ at higher pressure may result from the large deformation and possibly the densification of the porous structure. The excellent piezoelectric performance thus validated the design concept and confirmed the success of the fabrication process.

The analysis, as well as simulation studies from the literature [37], illustrated that three major structural parameters will affect the piezoelectric coefficient of the material: the honeycomb bubble width (a), the bubble height (b), and the bubble wall thickness (t). For this study, the bubble width was fixed at 3000 µm, and the effects of the bubble height and wall thickness on the sensitivity of the material were investigated. Precision clamping equipment was used during fabrication to accurately control the total thickness of the piezoelectric foam assembly and, therefore, the thickness of each bubble. The wall thickness of the bubble was studied by using COC films of different thicknesses for fabrication. An applied pressure of 4.9 kPa was used in the study of structure parameters on the sensitivity of the material. Figure 2b shows the effect of wall thickness on the sensitivity of the material. For piezoelectrets with the same bubble height, a larger wall thickness resulted in lower $d_{33}$. Doubling the wall thickness led to a reduction in $d_{33}$ of about 75–80%. The wall thickness significantly affected the elastic modulus of the material, and the thicker the bubble wall, the higher the elastic modulus [37]. As a result, the piezoelectric coefficient would decrease as the bubble wall thickness increases. Figure 2c shows the effect of the bubble height on the piezoelectric activity. An increase in bubble height led to a significant reduction in the piezoelectric activity. The bubble height affected the piezoelectric activity in two ways. First, a large bubble height resulted in a smaller aspect ratio and higher elastic modulus (Equation (2)), negatively impacting the piezoelectric activity. Furthermore, the increase in bubble height reduced the electrical field strength, required a higher breakdown voltage for charging, and resulted in a reduction in the surface charge density at the same charging voltage. The observed decrease in the piezoelectric activity was the compounded effect of the reduction in elastic modulus and the simultaneous decrease in surface charge density.

### 2.4. Thermal Stability of COC Piezoelectret

Figure 2d shows the results of a thermally stimulated discharge (TSD) measurement used to determine the thermal stability. The discharge peak was located at approximately 200 °C, with few discharges occurring when the temperature was lower than 150 °C. Further measurements showed that the quasistatic piezoelectric coefficient could remain approximately 90% of the original value after a thermal treatment at 150 °C for 30 min. The COC piezoelectrets exhibited excellent thermal stability, significantly better than PP piezoelectrets [15,16], the first and most commonly readily available material.

### 2.5. Study of the Behavior during the Charging Process and Actuation Behavior of the Material

**Figure 3:**
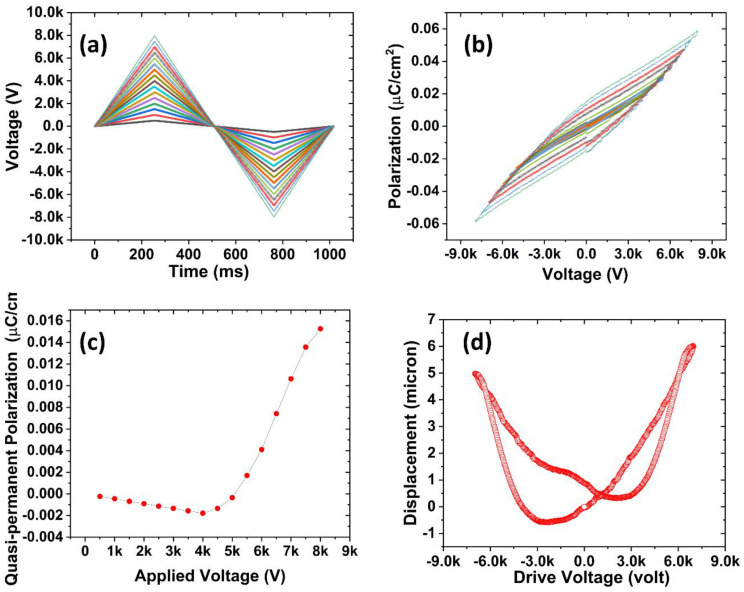
(**a**) AC-biased voltage applied to the material to study the charging process of the piezoelectric material. (**b**) Electrical hysteresis loop for the honeycomb structure during charging. (**c**) Quasistatic charge build-up inside the artificial void illustrated the charging breakdown voltage of the material. (**d**) Actuation behavior of the piezoelectric foam characterized by butterfly loop; sample applied with maximum voltage of 7000 V. Traces in different colors in (**a**,**b**) indicate applied charging voltages from 500 V to 8000 V in increments of 500 V.

The polarization behavior of the piezoelectrets was investigated using an electric hysteresis loop. Figure 3a shows the voltage profile, and Figure 3b shows the results from which the quasipermanent polarization can be extracted. Figure 3c shows the charge build-up in the pores during charging. The results show that the electrical dipole inside the artificial void started to build up with an applied voltage of around 4500–5000 V. The theoretical value for the honeycomb structure was evaluated using the established approach illustrated below. The breakdown of gas during the charging process follows Paschen’s law, for which the critical breakdown electric field strength is determined by:

(3) $$E_{bd}=\frac{ap}{\ln(pd)+b}$$

where *E_bd_* is the breakdown electric field strength, a is 4.36 × 10^7^ Vatm^−1^m^−1^, p is pressure (1 atm in the study), d is the thickness of gas (240 μm in the study), and b is equal to 12.8.

The charging threshold voltage was estimated using a layer model [38]:

(4) $$V_{bd}=\frac{ap}{\ln(ph_{air})+b}(\frac{h_{COC}}{\varepsilon_{COC}}+2h_{air})$$

where *ε_COC_* = 2.3 is the relative permittivity of COC; *h_air_* is the thickness of a single air bubble, which is equal to 240 μm in this investigation; *h_COC_* is the total thickness of the COC films, which is equal to 304.8 μm; and *V_bd_* is the breakdown threshold voltage. Substituting these parameters into Equation (4) yielded a value of 5980 V for the breakdown voltage. Although higher, the model predicted results that agreed reasonably well with the experimental values. The discrepancy might result from the model simplification, and possibly the slight reduction in the bubble height in the assembled structure.

**Figure 4:**
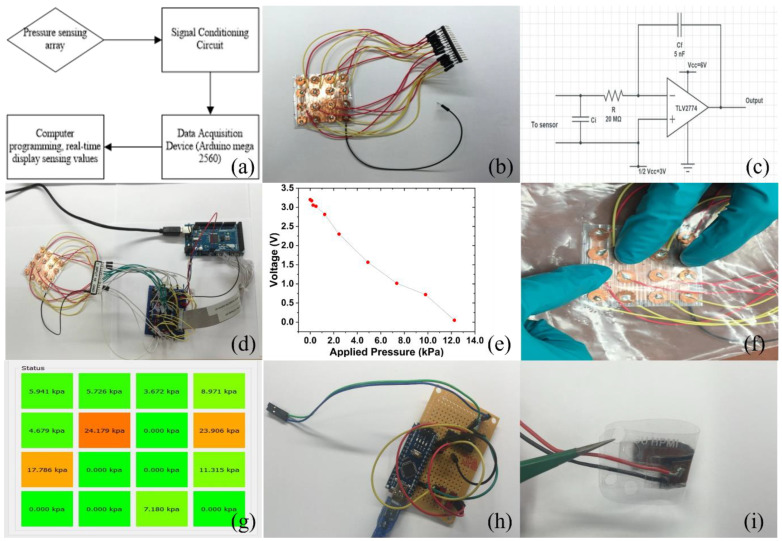
(**a**). Illustration of design of the pressure-sensing and mapping device based on COC piezoelectric materials; (**b**). Piezoelectric sensor signal-conditioning circuit converts high-impedance signals to low-impedance signals; (**c**). Voltage–pressure relationship of COC piezoelectric foam as the pressure-sensing result; (**d**). Photograph of the piezoelectric foam sensor array with 4 × 4 sensor for demonstration purposes; (**e**). Layout of the multipoint pressure-sensing device based on COC piezoelectric materials; (**f**,**g**). Demonstration of multi-point tactile-sensing capability of the material; (**h**). Demonstration of the capability of piezoelectric foam as flexible biosignal-measuring sensor; (**i**). A compact design of the sensing circuit demonstrated the wearable, low-cost, low-power-consumption pressure-sensing capability of the piezoelectric foam.

Below the breakdown voltage, no surface charge was accumulated inside the artificial void. When the applied voltage was higher than the threshold breakdown voltage, dielectric barrier discharge (DBD) occurred and charge started to build up in the artificial void [3]. Particularly, from Figure 4c, the quasipermanent charge was approximately linearly proportional to the applied voltage after the applied voltage reached the threshold breakdown voltage.

To study the actuation behavior of the material, a butterfly loop measurement was used to measure the displacement response to the applied electric field of the material. Figure 3d shows the results from which the inverse piezoelectric coefficient can be calculated to be 880 pmV^−1^, which is significantly higher than other piezoelectric materials such as PZT materials [39,40]. On the other hand, the inverse piezoelectric coefficient was lower than the quasistatic piezoelectric coefficient. This is different from common piezoelectric materials whose piezoelectric and inverse piezoelectric coefficient should be the same. Such behavior may arise from the difference in the responses of the structure element to mechanical and electrical excitation. During the mechanical quasistatic piezoelectric coefficient measurement, the mechanical stress was concentrated on the center of the honeycomb bubble, and the structure was easy to deform by flexing and/or rotation of the walls. By contrast, the stress was more evenly distributed during the actuation test because the surface charge was uniformly distributed by charging. Thus, the walls were evenly compressed with significantly lower strain.

### 2.6. Sensing Application of COC Piezoelectret

Pressure-sensing systems were designed and fabricated to demonstrate the pressure-sensing and mapping capabilities of the COC piezoelectric foam. Figure 4a shows a general design of the pressure-sensing system. Since the output signal of piezoelectric sensors is a high-impedance signal that cannot be measured with a standard multimeter, a charge amplifier (CMOS operational amplifier) was used to convert the signal to a low-impedance signal. Figure 4b shows the conversion circuit. The charge signal was connected to the gate side of the amplifier, and the output signal was the voltage. In the current design, the charge amplifier used was a TLV2774 from Texas Instruments. A power supply of 6 V was applied, and the bias voltage was set to 3 V. The output voltage was read with a common multimeter (Fluke 179). The charge amplifier was set to work in voltage mode because this mode can reduce the negative effect of the capacitance of connection cables on measurement in the whole circuit. The voltage output in regard to the input charge amount is described in Equation (5):

(5) $$V_{O}=-\frac{q}{c_{f}}+\frac{V_{cc}}{2}$$

where *V_o_* is the output voltage, *q* is the input charge, $c_{f}$ is the feedback capacitance, and *V_cc_* is the power supply voltage.

Figure 4c shows the result of the pressure–voltage relationship of a single piezoelectric sensor. An approximately linear relationship was observed between the applied pressure and output voltage with a coefficient of 0.258 VkPa^−1^ (R^2^ = 0.982). 

One unique advantage of piezoelectrets is that the entire surface is capable of sensing, and sensor arrays can be easily fabricated from a single piezoelectret with the simple placement of electrodes at select locations to extract signals. To demonstrate this, we fabricated a 4 × 4 sensor array from a single 3 × 3-inch piezoelectret. Figure 4d shows the sensor array, and Figure 4e shows the complete sensor system. A microcontroller (Arduino mega 2560) was used to collect the 16 channels of the analog voltage signal input from 0 V to 5 V. The system was connected to a computer via USB. Using an in-house-developed data acquisition algorithm, the data were displayed in real time on the position and value of the pressure signals. As a simple demonstration, select positions out of the 16 sensors were pressured simultaneously using fingers (Figure 4f), and the signals were properly detected and displayed at the 3 corresponding locations (Figure 4g). The sensor density in sensor arrays is determined by the number of electrodes one can integrate into the material, which can be significantly increased by applying other electrode-coating and fabrication techniques, or by integrating the MOSFET into the material itself [41]. This should further improve the sensing resolution. The pressure-sensing devices can also be fabricated to be very flexible (Figure 4h) and to have a small footprint (Figure 4i). They require a very low energy cost and are very lightweight. Compared to other pressure sensors, such as piezoresistive pressure sensors, the piezoelectric sensor consumes much less energy than a piezoresistive pressure sensors, as it itself serves as a charge source and does not require a supporting current to monitor the pressure. The working current of the single-channel sensor and its signal-conditioning circuit is theoretically less than 1 mA, which enables a single AA battery to power the sensing circuit for more than 3 months. 

**Figure 5:**
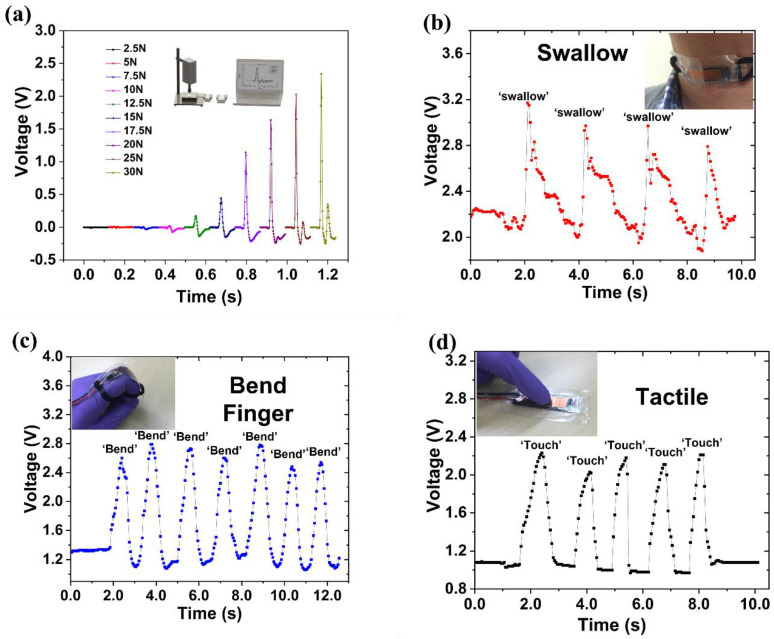
(**a**). The application of the COC piezoelectric sensor in measuring the impact events of real-time high-rate impacts. The sensing materials were placed on a soft PU foam support; (**b**). Real-time signal pattern of measuring the movement during swallowing, with the sensor directly attached to the tester’s neck; (**c**). The signal of the sensor in measuring the motion during finger bending; (**d**). Signal pattern corresponding to finger touch.

We next demonstrated the capability of the sensors to detect a variety of movements/motions encountered in many applications. Figure 5a illustrates the sensor response to impact forces. An impact tester was used to repeatedly strike the sensor with forces of different magnitude. The sensor performed well in detecting the impact events by almost instantaneously outputting sharp signal peaks of different magnitudes in real time. The sensor was also used to detect the movement of body parts. Figure 5b shows the sensor attached to a tester’s neck to monitor the swallow event of the person. As the tester swallowed, a signal was generated by the sensor with an instantaneous response. Figure 5c shows the detection of finger bending, which was well captured by the sensor. Figure 5d demonstrates the tactile-sensing capability of the sensor. Again, it performed well.

## 3. Conclusions

In this work, COC-based porous piezoelectric materials were developed based on a honeycomb structure that can be easily tuned by changing three design parameters. The design and fabrication of this structure address several key challenges towards achieving high piezoelectric activity with good structural stability. The piezoelectric materials from this design exhibited exceptionally high piezoelectric activity, with quasistatic piezoelectricity ranging from approximately 6000 to 12,900 pCN^−1^. The materials also showed excellent thermal stability, capable of maintaining good piezoelectric activity at a temperature of 150 °C. Using these materials, pressure-sensing systems were designed and constructed, and their pressure-sensing/mapping capabilities were demonstrated. The sensors also showed good performance in sensing a variety of motions/movements. With advantages such as excellent and tunable piezoelectric sensitivity, light weight, good flexibility, excellent thermal stability, low cost, low energy consumption, and ease of producing high-density sensor arrays, the developed materials may potentially be used in many applications involving sensing, actuating, energy harvesting, and many other fields.

## 4. Experimental Section

### 4.1. Fabrication of COC Piezoelectret

#### 4.1.1. Materials 

The COC film used in this study was TOPAS grade 6017 from TOPAS Advanced Polymers (Florence, KY, USA). Films of two different thicknesses of 50.8 µm and 101.6 µm were purchased.

#### 4.1.2. Structure Design

**Figure 6:**
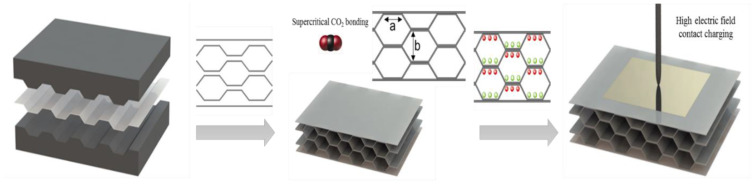
Schematic illustration of the fabrication procedure of the COC piezoelectric foam.

Figure 6 shows the schematic of the piezoelectric foam structure. The design employs a honeycomb structure with hexagonal channels as the structure unit with three tunable structure parameters: the width of the hexagon ‘a’, the height of the hexagon ‘b’, and the film thickness ‘t’. Variation in these parameters would result in different mechanical properties and charging characteristics on the piezoelectric foam.

#### 4.1.3. Fabrication of the Honeycomb COC Piezoelectric Foam

Figure 6 also illustrates the fabrication procedure of the COC piezoelectric foam. The overall process can be divided into two stages: the fabrication of the honeycomb structure and charging. The fabrication process of the honeycomb structure can also be divided into two steps: the preparation of the corrugated polymer film and the assembly of the honeycomb structure. 

In the first step, corrugated patterns were generated on the COC films. This was achieved through the compression molding of the flat films using a metal mold with premachined patterns. The compression molding was conducted by applying a controlled force of 10 N for 15 min at a temperature of 190 °C, slightly higher than the glass transition temperature of the COC polymer (178 °C). 

To fabricate the final honeycomb structure, four layers of the corrugated films and two flat outer layers were aligned, as shown in Figure 1, and fused together using a carbon dioxide assistant assembly [4]. The fusion and assembly were conducted in a pressure chamber for 20 h at a CO_2_ pressure of 10 MPa and a temperature of 120 °C, approximately 60 °C lower than the COC glass transition temperature (178 °C). 

In the final step, the assembled structure was coated with metal electrodes on both sides and charged using contact charging with a voltage of 8000 V.

### 4.2. Characterization of COC Piezoelectret

#### 4.2.1. Morphology

The morphology of the piezoelectric foam was characterized using an SEM and an optical microscope. The SEM used was JOEL JSM-7410F and the optical microscope was AM4112T-FV2W.

#### 4.2.2. Quasistatic Piezoelectric Coefficient

Quasistatic piezoelectric coefficient $d_{33}$ is a common parameter used to characterize the performance of piezoelectric material, especially piezoelectric foams, which is determined by:

(6) $$d_{33}=\frac{Q}{F}=\frac{\sigma }{p}$$

where *d_33_* is the quasistatic piezoelectric coefficient, *Q* is the amount of charge, *F* is the applied force, *σ* is the charge density, and *p* is the applied pressure.

In the study, the samples were measured by applying pressure from 0.245 kPa to 12.25 kPa. To eliminate the artifact caused by the airgap between the sample and electrode, a preload of 1.225 kPa was applied before introducing the measuring load. The charge induced under pressure was measured with an electrometer (Keithley 6517A).

#### 4.2.3. Thermally Stimulated Discharge–Current Spectra

Thermally stimulated discharge (TSD short-circuited) was used to study the thermal stability of the prepared materials. Both sides of the sample were sputter-coated and connected to an electrometer (Keithley 6517A). The temperature was increased at a rate of 3 °C min^−1^ from room temperature to the temperature at which the discharge current disappeared. The thermally stimulated current was measured with the electrometer.

#### 4.2.4. Electrical Hysteresis Loop and Butterfly Hysteresis Loop

To study the surface charge build-up process in the artificial void, an electric hysteresis loop test was used. The sample used in this characterization had a bubble thickness of 240 µm. Bipolar charging voltages from 500 V to 8000 V were applied during the hysteresis loop test. The characterization equipment used for the hysteresis loop and voltage–displacement butterfly hysteresis loop (butterfly loop) was the TREK model 609B and RADIANT Precision Premier II. The butterfly loop, which can characterize the material actuation behavior, was also measured with the electric hysteresis loop testing equipment. The samples were supplied with a bipolar drive voltage with a maximum value of 8000 V. The small deformations of the material were then measured with a high-precision optical fiber system (TF Analyzer 2000 system).

## Data Availability

Data available upon request to the authors.
